# Infection, Disease, and Transmission Dynamics in Calves after Experimental and Natural Challenge with a Bovine *Chlamydia psittaci* Isolate

**DOI:** 10.1371/journal.pone.0064066

**Published:** 2013-05-14

**Authors:** Carola Ostermann, Anke Rüttger, Evelyn Schubert, Wieland Schrödl, Konrad Sachse, Petra Reinhold

**Affiliations:** 1 Institute of Molecular Pathogenesis at ‘Friedrich-Loeffler-Institut’ (Federal Research Institute for Animal Health), Jena, Germany; 2 OIE (World Organisation for Animal Health) Reference Laboratory for Chlamydiosis at ‘Friedrich-Loeffler-Institut’ (Federal Research Institute for Animal Health), Jena, Germany; 3 Institute of Bacteriology and Mycology at the Veterinary Faculty, University of Leipzig, Leipzig, Germany; University of California Merced, United States of America

## Abstract

*Chlamydia* (*C.*) *psittaci* is the causative agent of psittacosis, a zoonotic disease in birds and man. In addition, *C. psittaci* has been repeatedly found in domestic animals and is, at least in calves, also able to induce respiratory disease. Knowledge about transmission routes in cattle herds is still deficient, and nothing is known about differences in host response after either experimental or natural exposure to *C. psittaci*. Therefore, our recently developed respiratory infection model was exploited to evaluate (i) the presence of the pathogen in blood, excretions and air, (ii) the possibility of transmission and (iii) clinical symptoms, acute phase and immune response until 5 weeks after exposure. In this prospective study, intrabronchial inoculation of 10^8^ inclusion-forming units of *C. psittaci* (n = 21 calves) led to reproducible acute respiratory illness (of approximately one week), accompanied by a systemic inflammatory reaction with an innate immune response dominated by neutrophils. Excretion and/or exhalation of the pathogen was sufficient to transmit the infection to naïve sentinel calves (n = 3) co-housed with the infected animals. Sentinel calves developed mild to subclinical infections only. Notably, excretion of the pathogen, predominantly via feces, occurred more frequently in animals naturally exposed to *C. psittaci* (i.e. sentinels) as compared to experimentally-inoculated calves. The humoral immune response was generally weak, and did not emerge regularly following experimental infection; however, it was largely absent after naturally acquired infection.

## Introduction

Psittacosis is a zoonotic disease caused by infection with the obligate intracellular bacterium, *Chlamydia* (*C.*) *psittaci*. The most important route of infection for humans and birds is inhalation of desiccated and aerosolized excreta (urine, feces, ocular, nasal, and respiratory tract secretions) of *C. psittaci*-shedding birds. Symptoms of both human and avian psittacosis encompass the whole range from clinically silent or mild flu-like symptoms to fatal disease [1]–[6]. Clinically inconspicuous cases of chronic infection in animals, where intermittent shedding of the pathogen continues, can easily be overlooked. Likewise, the frequency of reported human cases is thought to be underestimated [7], [8].

With the improvement of diagnostic tools during the last decade, *C. psittaci* was additionally found in various non-human mammals, such as sheep and pigs [9], [10], wild boar [11], horses [12], [13], dogs [14], [15] as well as in cattle [16]–[20]. Although chlamydial infections in cattle are mostly subclinical, they are very likely associated with reduced performance (e.g. retarded growth of calves, reduced fertility, and decreased milk yield [21], [22]). It was shown recently that experimental infection with a bovine *C. psittaci* strain was capable of inducing acute broncho-pneumonia in calves [23].

Chlamydiae are present in many bovine herds, but reports of cases involving farmers suffering from Chlamydia-caused respiratory disorders or asthma-like symptoms are only anecdotal and suggest the zoonotic potential being minimal compared to avian strains. While more definitive data on the identity and role of molecular virulence factors have been reported recently [24], [25], markers for the zoonotic potential of avian and non-avian strains of *C. psittaci* are still unknown. Analysis of complete genome sequences of mammalian *C. psittaci* isolates failed to identify a link between *C. psittaci* genotypes and host species [26]. It has been emphasized that all genotypes and serovars of *C. psittaci* should be considered potentially transmissible to humans [7], [27], [28].

Until now, routes of infection, shedding and transmission of the pathogen, as well as the long-term cause-effect relationships in bovine *C. psittaci* infection, have not been evaluated under standardized conditions, wherefore we exploited our recently introduced respiratory *C. psittaci* infection model in calves [23]. The aim of the present study was to obtain experimental evidence on routes and mechanisms of shedding, as well as transmission of the pathogen by socializing naïve sentinel animals with *C. psittaci*-challenged calves. Herein, the course of experimentally induced and naturally acquired infection will be evaluated with respect to clinical outcome, acute-phase reactions, and cellular vs. humoral immune response. Results of the present study support the validity of our hypotheses that (i) the pathogen is transmissible within bovine herds, (ii) *C. psittaci* is able to chronically impair health, and (iii) naturally acquired infections result in a milder course of disease compared to experimentally induced infection.

## Results

### Quantification of *C. psittaci* in blood and recovery from lung tissue

**Figure 1:**
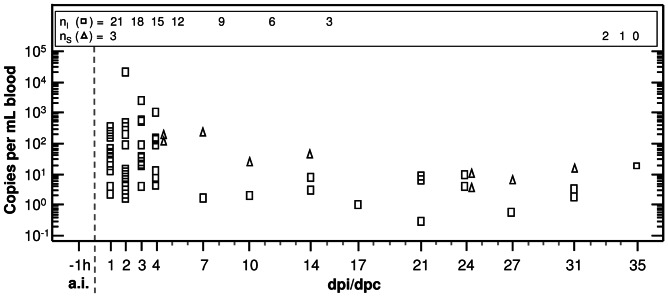
Detection of *C. psittaci* DNA in blood samples. The number of genomic equivalents to inclusion-forming units as determined by quantitative rtPCR was maximal within one week after both experimental challenge (squares) and natural acquisition (triangles). In experimentally challenged calves the maximal number of copies per mL blood varied between different animals to a large extent. Medians of three extractions, each run in duplicate were calculated for each blood sample. Mann-Whitney rank sum test was conducted to compare the amount of chlamydial DNA at different time points within the group of experimentally inoculated calves. Significant *P*-values are given in Table 1. a.i. *ante infectionem*. dpi days post inoculation. dpc days post contact to inoculated calves. h hour. n_I_ number of inoculated calves. n_S_ number of sentinels.

Examination of venous blood samples after challenge using real-time (rt)-PCR revealed that genomic DNA of *Chlamydiaceae* was found in the peripheral blood of 14 of 21 (67%) experimentally inoculated calves, and in two of the three sentinels. As depicted in Fig. 1, copy numbers per mL blood peaked within the first week after contact to the pathogen with variable maximal copy numbers. Statistical analysis confirmed that *C. psittaci* DNA contents were significantly higher in experimentally challenged calves compared to sentinels (Mann-Whitney rank sum test, *P* = 0.03). Detection of chlamydiae in blood was possible until the end of the study, i.e. 35 days post inoculation (dpi) in inoculated or 31 days post contact (dpc) in sentinels, respectively. Detailed information about significant, time-dependent differences in detected DNA amounts within the inoculated group is given in Table 1. DNA microarray examination of selected samples confirmed that indeed *C. psittaci* was found.

**Table 1:** Significant differences in the amount of chlamydial DNA in blood of calves challenged with 10^8^ ifu of *C. psittaci*.

Timepoint	1 dpi	2 dpi	3 dpi	4 dpi	7 dpi	10 dpi	14 dpi	17 dpi	21 dpi	24 dpi	27 dpi	31 dpi	35 dpi
−1h a.i.	***	***	***	***	-	-	*	-	***	***	-	*	-
7 dpi	**	**	**	**	-	-	-	-	**	*	-	*	-
10 dpi	*	*	**	**	-	-	-	-	**	*	-	-	-

Data are given in Figure 1.

Mann-Whitney rank sum test was used to compare the amount of copy numbers per mL blood at different time points. Significant differences are indicated by * *P*≤0.05, ** *P*≤0.01, *** *P*≤0.001. a.i. *ante infectionem*. dpi days post inoculation. h hour.

Recovery by cell culture of *C. psittaci* from lung tissue was successful in all experimentally challenged calves euthanized up to day 14. The identity of re-isolates to the challenge strain was exemplarily shown in four instances by sequencing of the ompA locus (data not shown). However, recovery from animals euthanized at day 35 was not successful, neither could the infectious agent be cultured from sentinels (necropsied >30 dpc).

### Detection of chlamydial DNA in excretions, exhaled breath and room air

None of the ocular, nasal or rectal swabs taken before inoculation or socialization were PCR positive for *C. psittaci*. Fecal shedding occurred in 5 of 21 inoculated calves (from 3 to 22 dpi) and in all sentinel calves (n = 3, from 5 to 21 dpc). On a cumulative basis, the proportion of positive rectal swabs in sentinels (13/60; i.e. 21.7%) exceeded the detection rate in experimentally challenged calves (positive rectal swabs: 8/152; i.e. 5.3%). However, comparison of the cumulated data is only legitimate for animals surviving up to the end of the study, i.e. 3 inoculated animals versus 3 sentinels. At the end of the study, the probability level of a higher fecal excretion in sentinels was 13,2% (Fisher's exact test). Comparing the groups at given time points confirmed a significantly higher detection rate in sentinels, compared to inoculated calves at 10 dpi (P≤0.05, Fisher's exact test). Later on, the group size of n = 3 was too small to obtain meaningful results. There was only one positive nasal swab at 6 dpi (1/153, i.e. 0.7%) in the inoculated group while all nasal swabs from sentinel calves were PCR negative. None of the ocular swabs in any group was positive.

All room air samples collected before challenge in either the animal rooms (n = 7) or the bronchoscopy room (n = 1) were negative for *C. psittaci*. From a total of 10 room air samples collected from the animal rooms (where the calves were housed after inoculation of *C. psittaci*), the two samples obtained at 1 and 2 dpi, respectively, were still negative, while 4 of 5 samples collected between 3 and 7 dpi were PCR positive. *C. psittaci* could not be detected anymore in the three room air samples collected 28, 30, and 35 dpi from the room where three remaining infected calves and three sentinels were co-housed.

In the three infected calves euthanized at 35 dpi, exhaled breath was screened for the presence of *C. psittaci* consecutively between 14 and 31 dpi. Only 1 of the 3 exhaled breath samples collected at 14 dpi was positive for the pathogen while *C. psittaci* could not be found in any of the samples collected at a later time point. Exhaled breath samples collected from sentinels between 22 and 29 dpc were also negative for *C. psittaci*.

### Clinical outcome

**Figure 2:**
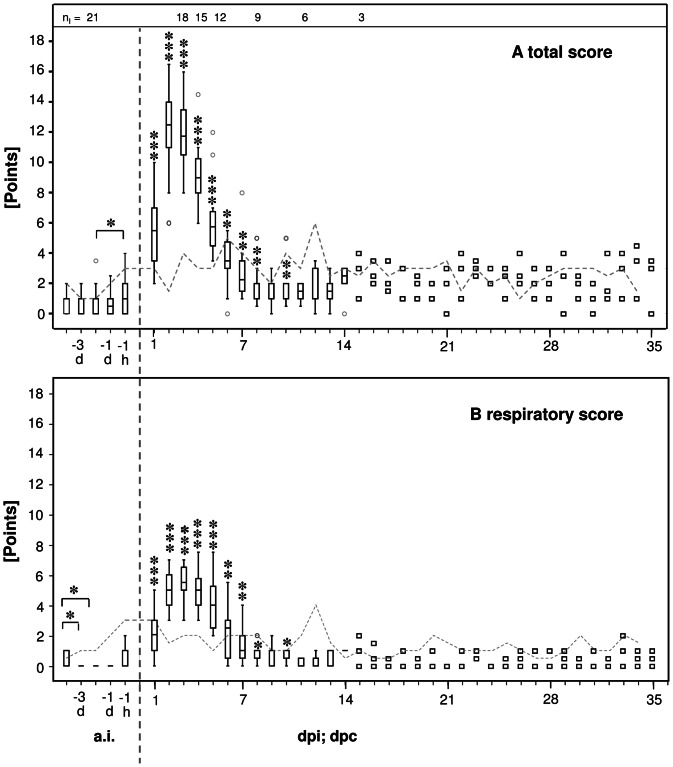
Score of clinical and respiratory health before and after *C. psittaci* challenge. General clinical score (panel A) and respiratory signs (panel B) peaked in the inoculated group 2–3 dpi. Symptoms resolved incompletely. The respiratory score contributed maximally 50% to the general clinical picture presented by the total score. In contrast, natural acquisition of *C. psittaci* infection lead only to mild symptoms in sentinel calves. Based on the group size of calves inoculated with 10^8^ ifu/animal (n_I_ given above panel A) data are given as Box and Whisker plots or as individual data (squares) if n≤3, respectively. The dashed line indicates the maximum of score points achieved in sentinels (n = 3). For statistical analysis, baseline data (averaged per animal) were compared to data obtained at different time points after challenge (Wilcoxon signed-rank test). Values of * *P*≤0.05, ** *P*≤0.01, and *** *P*≤0.001 were considered significant. Outlier values are indicated by small grey circles. a.i *ante infectionem*. d day. dpi days post inoculation. dpc days post contact to inoculated calves. h hour.

Inoculation of 10^8^ ifu of *C. psittaci* per calf resulted in acute respiratory illness, the signs of which were maximal at 2–3 dpi (Fig. 2A). From 4–8 dpi, health improved continuously, although none of the calves recovered completely by the end of this study. Both respiratory and total health scores at days 1 through 8 and day 10 after inoculation differed significantly compared to the mean values before inoculation (Wilcoxon signed-rank test, Fig. 2A, B). Acute clinical signs included elevated heart rates (max. 132 at 2 dpi), respiratory rates (max. 120 at 2 dpi), rectal temperatures (max. 41.6°C at 2 dpi), dry and scattered cough, reduced feed intake and hyperemic conjunctivae. Throughout the course of the study elevated respiratory rates, cough, hyperemic conjunctivae and enlarged mandible lymph nodes continued to occur.

Sentinels remained largely asymptomatic in terms of their general condition (e.g., appetite, conduct, and rectal temperature). The total and respiratory score did not exceed the sum of 6 out of 49 (Fig. 2A) or 4 out of 17 points (Fig. 2B), respectively. However, cough occurred between 7 to 11 dpc and continued intermittently up to the end of the study. Additionally, ocular or nasal discharge occurred, conjunctivae were often hyperemic, and mandibular lymph nodes were repeatedly enlarged between 15 and 29 dpc.

### Acute-phase response

**Figure 3:**
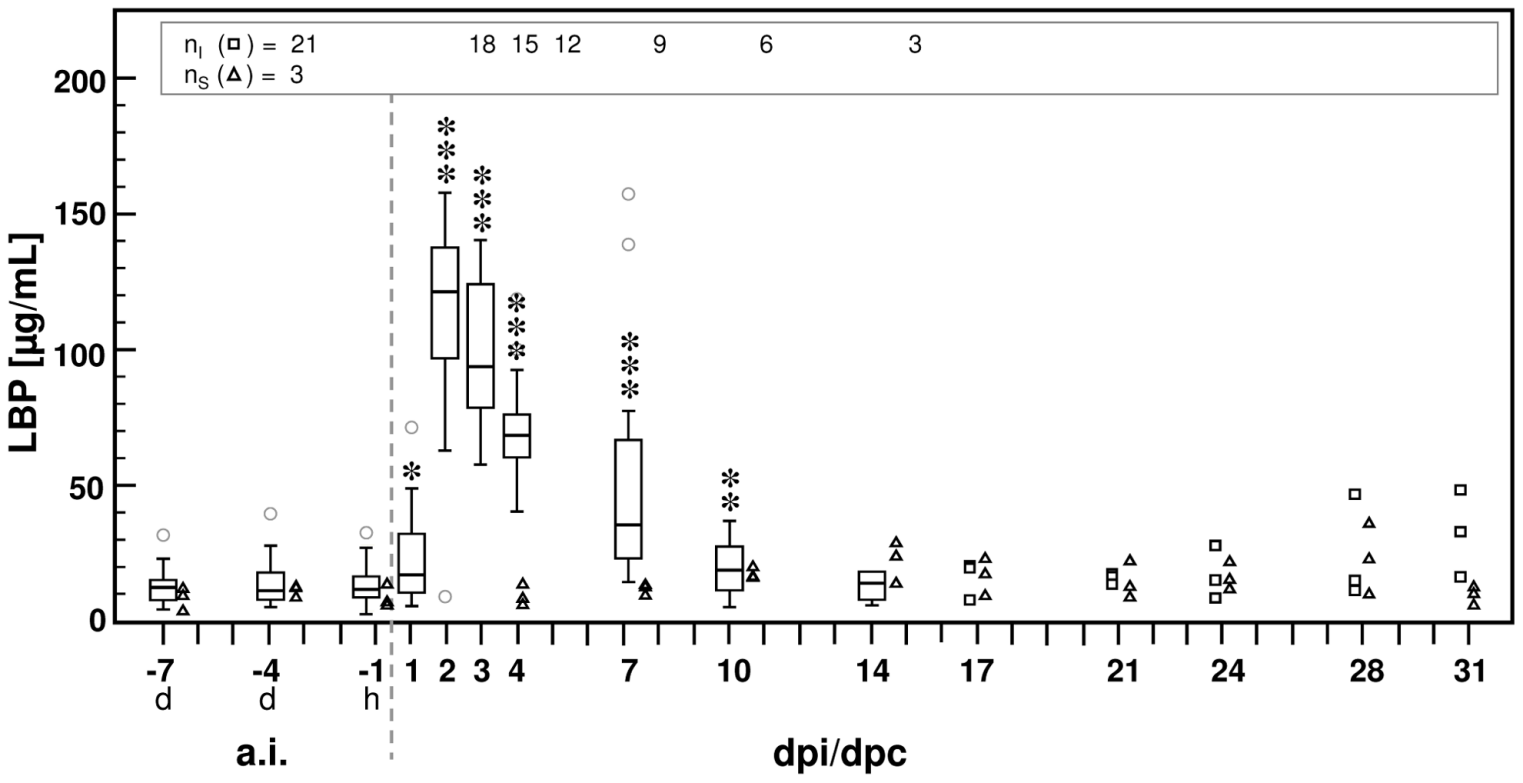
Concentration of lipopolysachharide binding protein (LBP) in peripheral blood before and after *C. psittaci* challenge. After inoculation of *C. psittaci*, LBP concentration was significantly increased until 10 dpi. Based on the group size of calves inoculated with 10^8^ ifu/animal (n_I_) data are given as Box and Whisker plots or as individual data (squares, if n≤3), respectively. The LBP concentrations obtained in sentinels (n_S,_ triangles) increased slightly after 7 days post contact (dpc) but did not exceed 35.1 µg/mL (i.e. maximal LBP concentration: at 28 dpc). Due to small sample size no statistical hypothesis was applied to this group. For statistical analysis of experimentally challenged calves, baseline data (averaged per animal) were compared to data obtained at different time points after challenge (Wilcoxon signed-rank test). Values of * *P*≤0.05, ** *P*≤0.01, and *** *P*≤0.001 were considered significant. Outlier values are indicated by small grey circles. a.i *ante infectionem*. d day. dpi days post inoculation. dpc days post contact to inoculated calves. h hour.

The concentration of lipopolysaccharide (LPS)-binding protein (LBP) in experimentally challenged calves rose from a stable baseline level before inoculation (median: 11.7 µg/mL, range: 37.0 µg/mL) to statistically significant elevated values between 1–10 dpi, with a maximum at 2 dpi (median: 121.3 µg/mL, range: 148.8 µg/mL). Towards the end of the study, individual LBP concentrations remained elevated compared to median baseline values before inoculation (Fig. 3).

In sentinels, the concentration of LBP increased between 10–28 dpc, but this increase was transient and mild compared to inoculated calves (Fig. 3).

### Systemic cellular response

#### White blood cells in inoculated calves

The total number of peripheral blood leukocytes (Table S1) increased within the first two days after inoculation and was maximal and significantly elevated at 2 dpi. After dropping below baseline level at 3 and 4 dpi, the number of leukocytes measured between one and two weeks after challenge revealed an ascending tendency, which was significant at 14 dpi compared to baseline data. Individual leukocyte numbers between 17–35 dpi also tended to exceed the pre-inoculation median in the majority of calves.

**Figure 4:**
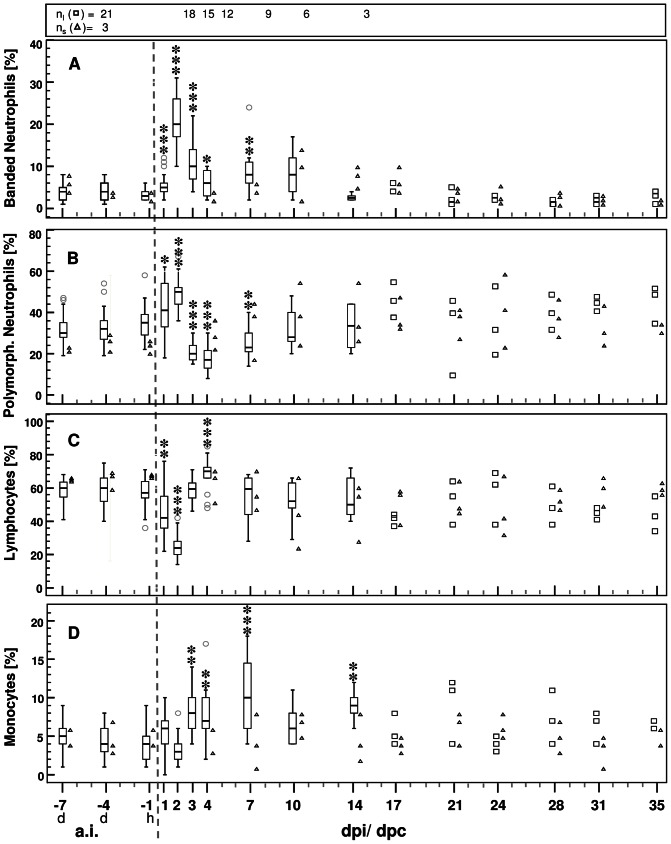
Relative amounts of white blood cells before and after *C. psittaci* challenge. Compared to baseline data, both banded and polymorphnuclear neutrophils increased during the acute phase of infection whilst lymphocytes decreased transiently. Based on the group size of calves inoculated with 10^8^ ifu/animal (n_I_) data are given as Box and Whisker plots or as individual data (squares, if n≤3), respectively. For statistical analysis, baseline data (averaged per animal) were compared to data obtained at different time points after challenge (Wilcoxon signed-rank test). Values of * *P*≤0.05, ** *P*≤0.01, and *** *P*≤0.001 were considered significant. Outlier values are indicated by small grey circles. Due to small sample size of sentinel calves (n_S,_ triangles) no statistical hypothesis was applied to this group. a.i *ante infectionem*. d day. dpi days post inoculation. dpc days post contact to inoculated calves. h hour.

The early increase of leukocytes in inoculated calves was mainly driven by an increase of both mature and band forms of neutrophils, which where maximal at 2 dpi on a percentage basis and based on absolute numbers (Fig. 4 A, B, Table S1). The absolute number of polymorphonuclear neutrophils as well as their relative amount dropped sharply at 3 dpi and median values were below the initial medians up to 7 or 10 dpi, respectively. From 7 dpi towards the end of the study, the relative number of polymorphonuclear neutrophils tended to rise again and individual values exceeded mostly the initial level at the last days of the study (Fig. 4 B). In contrast, banded neutrophils exceeded the baseline value significantly up to 10 dpi (except at 4 dpi) and returned back to initial levels towards the end of the study (Fig. 4A, Table S1).

The absolute amount of lymphocytes was slightly but significantly reduced between 1–4 dpi and subsequently returned to the initial level (Table S1). On a percentage basis, the clear reduction of lymphocytes at 1 and 2 dpi was followed by an upward trend and a statistically significant maximum at 4 dpi (Fig. 4C). At the final stage, individual values of lymphocytes were generally lower compared to initial values on a percentage basis.

The proportion of monocytes was significantly elevated on 3, 4, 7, and 14 dpi (Fig. 4D). Absolute and relative amounts of monocytes measured hereafter tended to be elevated compared to initial values (Fig. 4D and Table S1).

#### WBCs in sentinel calves

In sentinels, the total number of blood leukocytes increased constantly up to 10 dpc (individual maximum of 19.25 G/L). Towards the end of the study, they slowly approached the initial level (Table S1). The absolute number of polymorphonuclear neutrophils was clearly elevated between 10–21 dpc and did not reach the initial level up to the end of the study. On a percentage basis, polymorphonuclear neutrophils were elevated in the final period with individual maxima on 7, 24 and 28 dpc (44, 58 and 46%, Fig. 4B). Banded neutrophils peaked between 10–17 dpc in both absolute and relative numbers and returned to initial levels afterwards (Table S1, Fig. 4A). Absolute numbers of lymphocytes and monocytes did not show clear trends up to the end of the study.

### Humoral immune response

Western immunoblotting revealed the formation of specific antibodies against the *C. psittaci* challenge strain DC15 in four of the six inoculated calves that were euthanized at 14 and 35 dpi. The onset of the specific humoral immune reaction has been detected between 10 and 14 dpi. In one calf, the antibody level was maintained until the end of the trial whereas in two other calves, antibodies decreased towards the end of the study (data not shown). In sera of sentinel calves, no chlamydia-specific antibodies could be detected at any time point.

## Discussion

### Horizontal transmission of *C. psittaci*

In the present experimental setting of intrabronchial inoculation of 10^8^ ifu *C. psittaci* per calf, clinical signs were induced, bacteremia occurred, pathogens were recovered from lung tissue, and humoral immune response revealed successful infection. The excretions of inoculated calves were sufficient to transmit infection to naïve sentinels as indicated by the presence of the pathogen in their peripheral blood. To the best of our knowledge, this is the first study emulating natural animal-to-animal transmission of *C. psittaci* in bovine herds. More importantly, these findings substantiate the hypothesis that, since *C. psittaci* infection was easily transmissible from calf to calf, humans in close contact with infected animals could similarly be at risk of exposure.

Interestingly, less than one fourth of the calves excreted DNA of the pathogen after experimental infection, but all of naturally contracted sentinel calves did. In both groups, fecal excretion was predominant compared to nasal and/or ocular secretion or exhalation. There are two hypotheses to explain the presence of *C. psittaci* DNA in feces. (i) Mucociliary clearance transported the pathogen to the pharynx where it was subsequently swallowed. As such, *C. psittaci* might pass into the gastrointestinal tract without further interaction or might instead infect the mucosal cells. (ii) The pathogen was spread by bacteremia to the intestinal mucosa, where it infected epithelial cells that were released during the epithelial turn-over into the gastrointestinal tract. Findings of our study provide strong evidence for the first hypothesis because *C. psittaci* DNA was detected in tracheal mucus, but not in epithelial cells of upper airways neither in the intestinal mucosa [29].

Detection rates in rectal swabs of sentinels were four-fold higher compared to inoculated calves indicating that the route of infection might influence the shedding. Aerosolization of dried feces and contaminated dust is considered to be the most common transmission route of psittacosis originating from birds [8]. In the present study, despite complete daily cleaning of the environment, *C. psittaci* was routinely detected in room air during the acute phase of infection (3–7 dpi). In addition, the pathogen was sporadically detectable in exhaled breath even 14 dpi, i.e. at a time point when acute clinical signs had largely dissipated. Although inhalation of bio-aerosols is a likely route of air-borne transmission, alternative possibilities should be taken into consideration. Routes of zoonotic transmission from birds to man include ingestion of contaminated feces, mouth-to-beak contact, bites, open wounds, and blood-sucking ectoparasites [30]. In the present study, transmission via vectors (e.g., insects, parasites) can be ruled out, but group housing with direct contact certainly opened ways of direct (e.g. fecal-oral) transmission through mutual licking. It was reported that the risk of infection with *C. pecorum* and *C. abortus* in calves increased in a quadratic regression with group size, even though calves had physical contact only with their direct neighbours [31].

### Chlamydemia

Hematogenic spread of the pathogen has been shown by quantitative rt-PCR after both experimentally induced and naturally acquired infection. In inoculated calves euthanized at 35 dpi, low levels of chlamydial DNA were detectable in peripheral blood up to the end of the study, which suggests an ongoing systemic presence of the pathogen. Although rt-PCR neither allows identification of cell types carrying chlamydiae nor assessment of the viability of the pathogen, we assume that *C. psittaci* behaved similarly as described for *C. pneumoniae* in murine macrophages in vivo [32], where the agent was capable of infecting, surviving and multiplying. While macrophages enabled the dissemination of viable chlamydiae to distant tissue sites, DNA of initially inactivated chlamydiae in macrophages was rapidly degraded. Furthermore, *in vitro* studies revealed an improved long-term survival in macrophages after presence in apoptotic neutrophils [33]. Last but not least, cell-independent transport of elementary bodies (EBs) in blood cannot be excluded, as direct binding of *C. trachomatis* and *C. pneumoniae* EBs to apolipoprotein B containing fractions of plasma lipoproteins (i.e. low and very low density lipoproteins) was shown *in vitro* [34].

### Pathogenesis

#### Clinical outcome

Both character and intensity of clinical signs observed in the 21 calves inoculated with 10^8^ ifu of *C. psittaci* were consistent. Thus, the results provide statistically meaningful evidence confirming the general reproducibility of this particular dose in an animal model introduced recently by our group using different doses (10^6^–10^9^ ifu) [23].

The acute clinical outcome in inoculated calves was maximal between 2 and 3 dpi, and lasted for about one week. During acute illness, the total health score comprised up to 50% signs related to the respiratory score, which indicates strong involvement of the respiratory system. Thus, clinical findings in experimentally infected calves are in accordance with the few cases of acute respiratory disease due to *C. psittaci* infections reported in calves [17], [35].

Sentinels acquired the infection, but remained almost clinically inconspicuous. Although obvious signs of illness were lacking in these animals, the few mild clinical signs observed with a certain time delay were comparable to the low clinical score values seen in inoculated calves in the period from 14 dpi onwards. These observations support previous findings revealing the negative impact of naturally acquired subclinical *Chlamydia* spp. infections on calf health [36]. Furthermore, observations in the field indicated that the vast majority of widely spread chlamydial infections in cattle remain asymptomatic, but show a clear association to reduced growth rates in calves [22], and performance losses in dairy cows [20], [21].

#### Acute phase and innate immune response

Even the clinically inconspicuous acquisition of the pathogen in sentinel calves resulted in a slight increase of LBP concentration in blood. In calves experimentally challenged with *C. psittaci*, LBP concentrations in peripheral blood were markedly elevated during the acute phase which is in good agreement with LBP concentrations measured previously in blood of calves challenged with an identical dose of either live or inactivated *C. psittaci* [23]. Taking these findings together, LBP can be regarded as a reproducible and dose-dependent LPS-related biomarker in bovine chlamydiosis. Although the functional consequence of an LBP increase remains to be elucidated, our findings extend the relevance of LBP which was previously described as a fast-reacting sensitive marker of bovine respiratory disease [37], [38], [39].

In experimentally challenged calves, the leukocytic response of the innate immune system was prompt. Leukocytosis (i.e. >12.0 G/L; [40]) at 2 dpi was mainly driven by the increase of both polymorphonuclear and banded neutrophils, i.e. regenerative left shift in the leukocyte curve. This rapid, distinct increase of neutrophils was followed by a subsequent drop that can be interpreted as recruitment to the site of infection. This interpretation is in accordance with our previous findings concerning fibrinopurulent bronchopneumonia and influx of neutrophils into the lung at 3 dpi [23], as well as with several studies reporting the rapid recruitment of neutrophils in Chlamydia-induced pneumonia [41], [42], [43], [44].

Although acute symptoms subsided, the demand for leukocytes in blood remained elevated towards the end of the study, thus indicating again the shift from the acute phase into a subclinical persisting course. During this subclinical phase, neutrophils and monocytes tended to be elevated on a percentage basis, while relative amounts of blood lymphocytes were lowered, which corresponds in general to the composition of blood cells in naturally chlamydia-infected young cattle [36].

Even though we used Western immunoblotting to test the calf sera, which is more sensitive than currently available enzyme linked immunosorbent assays (ELISA), only two thirds of experimentally challenged calves and none of the sentinels developed chlamydia-specific antibodies. This indicates that the presence of *C. psittaci* DNA in blood was not associated to humoral immune response induction. This observation is not surprising as previous studies demonstrated that only about 60% of naturally *C. abortus* and/or *C*. *pecorum* infected calves were sero-positive for *Chlamydia* spp. [36] and that seropositivity and chlamydial shedding do not necessarily correlate [18], [45]. Both human and veterinary medicine are faced with the problem that chlamydial infections often do not elicit sufficiently high antibody responses, which complicates both development of specific serological tests and development of safe and efficacious vaccines [46].

## Materials and Methods

### Ethics statement

This study was carried out in strict accordance with European and National Law for the Care and Use of Animals. The protocol was approved by the Committee on the Ethics of Animal Experiments and the Protection of Animals of the State of Thuringia, Germany (Permit Number: 04-002/07). All experiments were done in a containment of biosafety level 2 under supervision of the authorized institutional Agent for Animal Protection. Bronchoscopy to inoculate the pathogen was strictly performed under general anesthesia. During the entire study, every effort was made to minimize discomfort or suffering.

### Animals

Calves originated from a conventional farm without any history of *Chlamydia*-associated health problems. All animals (n = 24) were male and except for one, they all belonged to the Holstein Frisian breed. They were purchased at the age of 14 to 25 days (19.0±3.2, mean±SD) weighing between 46.2 and 71.2 kg (57.1±5.5, mean±SD). After arrival they had a quarantine period of at least four weeks (31±3.2 days, mean±SD).

The study was carried out in the Federal Research Institute for Animal Health (Friedrich-Loeffler-Institut, Jena, Germany) where animals were reared under standardized conditions (completely daily cleaning, room temperature: 18–20°C, relative humidity: about 60%) and in accordance with international guidelines for animal welfare. Feeding included hay and water ad libitum and individual supplementation with commercial milk replacer and coarse meal. All food products were free from antibiotics.

#### Exclusion of co-infections

The herd of origin was known to be free from bovine herpes virus 1 (BHV-1) and bovine virus diarrhoea/mucosal disease virus (BVDV). The latter status was confirmed, using BVDV-specific immunohistochemistry of ear biopsies [47]. Serological findings (Bio-X respiratory penta ELISA Kit, Bio-X-Diagnostics) confirmed that animals did not acquire infections with respiratory viruses (i.e. bovine respiratory syncytial virus (BRSV), parainfluenza 3 virus (PI-3) or adenovirus type 3) during the study. To verify the absence of bacterial co-infections (e.g., *Mycoplasma* (*M*.), *Pasteurella* (*P.*), *Mannheimia spp*.), nasal swabs were taken (i) before challenge and (ii) before necropsy. In addition, lung tissue was obtained during necropsy, but neither *P. multocida* nor *M. bovis* were detected in any sample. Colonisation of *Mannheimia haemolytica* was found in two calves euthanized at 2 and 4 dpi (once in lung tissue and nasal swab and once only in a nasal swab) without pathological consequences. In addition, nasal swabs were sporadically positive for *P. multocida* and *M. bovirhinis* (i.e. 4.3% and 14.9% respectively), both are known commensals of the nasal mucosa of healthy calves [48], [49]. In two instances, further non-pathogenic agents (*Acholeplasma laidlawii* and *M. canadense*) were detected from nasal swabs. Calves were also checked and found to be negative for the presence of *Salmonella* spp. (via rectal swabs).

### Study design

After the quarantine period and confirmation of physical health, all 24 calves aged 42 to 58 days (50.1±3.7 days, mean±SD) and weighting 59.2 to 93.0 kg (74.4±7.2 kg, mean±SD) were included. The entire study lasted from one week before challenge until 5 weeks after exposure.

**Figure 5:**
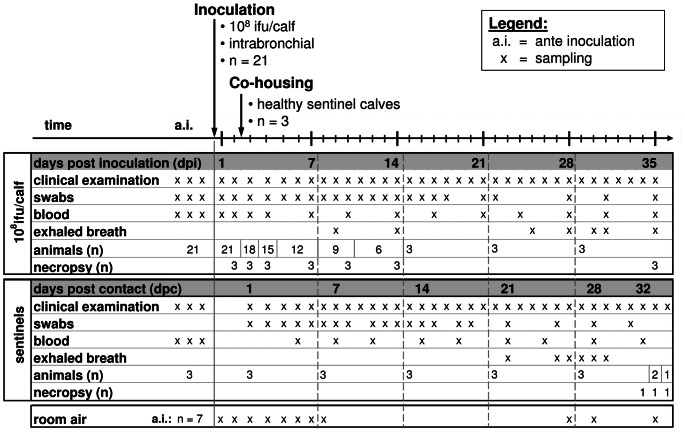
Study design. During this prospective study, three samples were collected before experimental or natural challenge with *C. psittaci* and hereafter on time points given below the timeline for both study groups.

Twenty-one calves were challenged intrabronchially with 10^8^ ifu of a bovine *C. psittaci* strain (DC15) as described elsewhere [23], and were housed in three communicating rooms (n = 7 per room). Three calves served as naïve sentinels, co-housed with the experimentally infected ones by replacing those animals euthanized at 2 dpi. Further necropsies of three calves per time point were performed at 3, 4, 7, 10, and 14 dpi (Fig. 5). After 14 days, the remaining 3 experimentally inoculated calves and the 3 sentinels were housed in one room until the end of the study. While the 3 inoculated calves were euthanized 35 dpi, the sentinels were euthanized at 32, 33, and 34 dpc. All necropsies were performed as described previously [23].

At time points depicted in Figure 5, in vivo sampling included (i) ocular, nasal and rectal swabs for detection of the pathogen and (ii) collection of peripheral blood from the jugular vein in order to asses acute phase response, immune response, and bacteremia. Swabs and blood were generally collected before morning feeding.

### Quantitation of *Chlamydiaceae* from blood samples using rt-PCR and species detection

Blood of all calves was tested before and after challenge according to time points given in Fig. 5. Therefore, aliquots of 300 µl of each blood sample were DNA extracted using the Qiagen Stool Mini Kit (Qiagen, Hilden, Germany) according to the instructions of the manufacturer. Detection of *Chlamydiaceae* spp. DNA was done on a Mx3000P (Agilent, Waldbronn, Germany) using a TaqMan rt-PCR protocol targeting the 23S rRNA gene with an internal amplification control as described previously [50], [51]. Amplification reactions contained 5 µl of DNA extract and were conducted on 96-well plates. A decimal dilution series of defined contents of inclusion-forming units (10^4^–10^−1^ ifu of *C. psittaci* strain DC15) was included in each run, and the resulting calibration curve served as the basis for copy calculation by the software MxPro 4.1 (Agilent, Waldbronn, Germany). Three extractions of each sample were tested in duplicate. For further statistical analyses the median of all six runs was used per calf and time point. For identification of the chlamydial species, selected *Chlamydiaceae* positive samples were examined by use of DNA microarray test [52] and a rt-PCR assay for *C. psittaci* [51].

### Recultivation of the pathogen from lung tissue and strain identification

For recovery of *C. psittaci*, lung tissue with inflammatory alterations (if present) obtained during necropsy was immediately placed in sterile transport medium for *Chlamydia* spp. (SPGA [53]), and further processed using standard procedures. Briefly macerated and homogenized tissue was used to inoculate BGM cell layers, and chlamydial inclusions were detected by immunofluorescence. For strain identification a 1088-bp segment of the ompA gene comprising all variable domains was sequenced as described [54].

### Confirmation of *C. psittaci* in swabs using rt-PCR

Ocular, nasal and rectal swabs were DNA extracted as described previously [55]. All swab samples were run in duplicate using the same rt-PCR methodology [50]. To confirm the presence of *C. psittaci*, *Chlamydiaceae*-positive samples were further examined using species-specific rt-PCR [51]. Selected *C. psittaci* positive samples where confirmed using DNA microarray tube assay [52].

### Sampling and analysis of room air and exhaled breath

Stable air was collected as recommended in literature [56] using an AirCheck XR 5000 instrument with standard IOM personal inhalable dust sampler (both SKC Inc., Eighty Four, PA, USA), which was equipped with gelatine filters of 3 mm pore size (SKC Inc., Eighty Four, PA, USA). The instrument was placed out of reach for the calves and pumped a total of 480 L through the filter at a flow rate of 2 L/min.

To analyze exhaled breath, the gelatine filter assembled IOM sampler was wired to the expiratory side of a Y-shaped inspiratory–expiratory valve, which was adapted to a tightly fitting face mask for calves (Kruuse, Langeskov, Denmark) as shown in Fig. S1. For each exhaled breath collection, the calf in- and expired through the mask for one hour.

Samples of both room air and exhaled breath were collected at time points given in Figure 5. For further PCR analyses, gelatine filters were processed and DNA extracted using the High Pure PCR Template Preparation Kit (Roche Diagnostics, Mannheim, Germany) according to the instructions of the manufacturer. To confirm the presence of *C. psittaci*, *Chlamydiaceae*-positive samples were further examined using species-specific rt-PCR [51].

### Clinical examination and scoring

Starting 4 days before challenge, clinical examination of each animal was performed daily until the end of the study. Results were summarized using a score system. Criteria for evaluation of clinical signs and the corresponding scoring system have been described elsewhere [23]. Shortly, the total health score consisted of sub-scores reflecting different organ systems. For the respiratory score, breathing frequency, occurrence of dyspnoea, ocular and nasal discharge, and the presence and inducement of cough were taken into account.

### Analysis of lipopolysaccharid-binding protein (LBP)

Venous blood was sampled into 9.0 mL plastic syringes (S-Monovettes, Sarstedt AG & CoKG, Nuembrecht, Germany) and serum was harvested by centrifugation (3120 g; 15 minutes, 15°C). Sera were stored at −80°C until analyzed. Concentrations of LBP were measured using an ELISA as described previously [57].

### White blood cell analysis

For white blood cell count (WBC), 20 µl of the collected potassium EDTA blood were prepared using Leuko–tic kit (bioanalytic GmbH, Umkirch/Freiburg, Germany). A Neubauer-improved hemoxymeter was filled, and WBSs were counted under 100-fold magnification of a bright light microscope. For cell differentiation, air dried blood smears were stained with HemaDiff - Quick Staining Set (bioanalytic GmbH, Umkirch/Freiburg, Germany). One hundred WBCs were differentiated under 400 fold magnification and served as basis to calculate absolute numbers of different cell types.

### Antigen preparation, SDS-PAGE and immunoblot

Immunoblotting was performed as described previously [23] for all animals which were included in the study for at least 14 days, i.e. six inoculated animals and all sentinels. Time points of blood sampling are given in Figure 5. Sera were harvested by centrifugation as described above, and stored at −20°C until analyzed.

### Statistical methods

SPSS (Version 19.0, IBM Corporation, NY, USA) and Statgratgraphics Centurion XVI (StatPoint Technologies, Inc., VA, USA) were used for statistical evaluation of the data. Normal distribution was tested using Kolmogorov-Smirnov Goodness-of-Fit Test. As the majority of data was not normally distributed, Wilcoxon signed-rank test was carried out to compare baseline data to daily post-inoculation values for the group of inoculated calves. In ‘Box and Whisker plots’, outlier values (circles) are 1.5–3 times of the length of a box away from the median.

Due to the small number of sentinels (n = 3) a within group approach was not possible. Descriptive data are given as minimum and maximum. Comparison of sentinels to the inoculated group, (per time point or compared to the three inoculated calves surviving to the end of the study) were performed by means of Mann-Whitney rank sum test or by Fisher's Exact test for categorical data (i.e. qualitative PCR analysis of fecal swabs). In case of quantitative rt-PCR of blood, medians of three extractions, each run in duplicate were compared at different time points using Mann-Whitney rank sum test. Generally values of * *P*≤0.05, ** *P*≤0.01, and *** *P*≤0.001 were considered significant.

## Supporting Information

Figure S1 **Sampling of Exhaled Air.** For sampling of exhaled air, calves wore a tightly fitting face mask which was adapted to a Y-shaped inspiratory–expiratory valve. Each animal inspired for one hour through the inspiratory valve (IN) and expired through the expiratory valve (EX) towards the IOM-Sampler. The IOM sampler was assembled with a gelatine filter, which was subsequently DNA extracted and PCR analyzed.
(TIF)

Table S1 **Absolute cell counts of white blood cells.** The amount of white blood cells is given as median [minimum; maximum] for the inoculated group and as [minimum; maximum] for sentinels (n  =  3). Baseline values (mean of data before inoculation) were tested against post inoculation values by means of Wilcoxon signed-rank test. Values of ^A^ P≤0.05, ^B^ P≤0.01, and ^C^ P≤0.001 were considered significant. Arrows indicate significant increase (↑) or decrease (↓) compared to baseline data. dpi days post inoculation. dpc days post contact to inoculated calves. No statistical hypothesis was applied to the sentinel group.
(XLS)
