# Supplementary figures and images for: Infection, Disease, and Transmission Dynamics in Calves after Experimental and Natural Challenge with a Bovine Chlamydia psittaci Isolate
Source: PLoS One. 2013 May 14;8(5):e64066. doi: 10.1371/journal.pone.0064066 (PMC3653844; doi:10.1371/journal.pone.0064066)

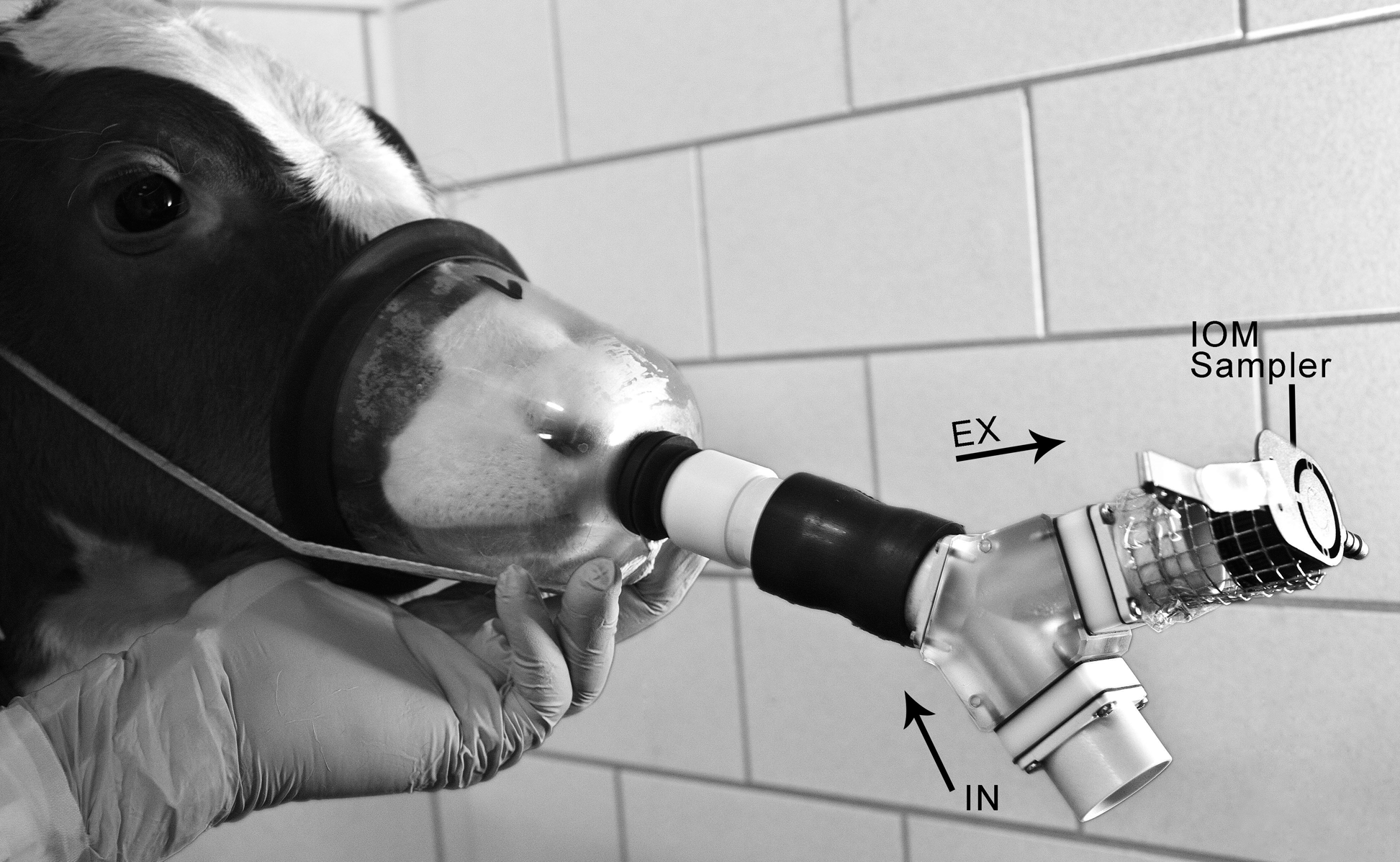

Supplement: Figure S1 — Sampling of Exhaled Air. For sampling of exhaled air, calves wore a tightly fitting face mask which was adapted to a Y-shaped inspiratory–expiratory valve. Each animal inspired for one hour through the inspiratory valve (IN) and expired through the expiratory valve (EX) towards the IOM-Sampler. The IOM sampler was assembled with a gelatine filter, which was subsequently DNA extracted and PCR analyzed. (TIF) [file pone.0064066.s001.tif]
